# Fibromyalgia: When Distress Becomes (Un)sympathetic Pain

**DOI:** 10.1155/2012/981565

**Published:** 2011-09-19

**Authors:** Manuel Martinez-Lavin

**Affiliations:** Rheumatology Department, National Institute of Cardiology, Juan Badiano 1, 14080 Mexico City, DF, Mexico

## Abstract

Fibromyalgia is a painful stress-related disorder. A key issue in fibromyalgia research is to investigate how distress could be converted into pain. The sympathetic nervous system is the main element of the stress response system. In animal models, physical trauma, infection, or distressing noise can induce abnormal connections between the sympathetic nervous system and the nociceptive system. Dorsal root ganglia sodium channels facilitate this type of sympathetic pain. Similar mechanisms may operate in fibromyalgia. Signs of sympathetic hyperactivity have been described in this condition. Genetic factors and/or distressful lifestyle may lead to this state of sympathetic hyperactivity. Trauma and infection are recognized fibromyalgia triggers. Women who suffer from fibromyalgia have catecholamine-evoked pain. Sympathetic dysfunction may also explain nonpain-related fibromyalgia symptoms. In conclusion, in fibromyalgia, distress could be converted into pain through forced hyperactivity of the sympathetic component of the stress response system.

## 1. Introduction

The key issue in fibromyalgia (FM) research is to define why people suffering from this illness have so much pain. FM is a stress-related disorder [1]. Patients who have FM often associate the onset of their illness to a particularly stressful situation such as physical or emotional trauma [2–4] or to different types of infections [5]. Additionally, they are frequently immersed in a distressful life style [6]. This article reviews scientific evidence suggesting that, in FM, distress becomes pain through malfunction of the sympathetic component of the stress response system. The following topics will be analyzed.

- Definition of stress, distress, and allostasis.
- The sympathetic nervous system as a key element of the stress response system.
- The autonomic nervous system as a complex adaptive system.
- Animal models linking the development of sympathetic pain to physical or emotional trauma and to different types of infections.
- Dorsal root ganglia sodium channels as key elements in sympathetically maintained pain.
- Physical and emotional distress in FM.
- Genetic and clinical data suggesting that FM is a sympathetically maintained neuropathic pain syndrome.
- Conclusions.

## 2. Stress, Distress, and Allostasis

The term stress is used in various ways and has different interpretations. Stress has been used to describe the cause (stressor) or the effect (stressed) of a phenomenon. An acceptable physiological definition of stress could be “any stimuli, physical or emotional, that threatens homeostasis” [7]. The term distress is also ambiguous. It was coined by Selye to describe maladaptive responses to stress leading to somatic and/or psychological harm [8]. The emotional component of distress is manifested as anxiety, exhaustion, and/or depression. There are other medical terms related to stress such as allostasis (the additional effort necessary to maintain equilibrium) and allostatic load (the price the body pays for being forced to adapt to adverse psychosocial or physical situations). These terms may also be pertinent in the study of FM pathogenesis [9]. Stress, distress, and allostasis have both physical and emotional connotations. Novel approaches to understanding complex diseases such as FM steer clear from the Cartesian mind-body dualism, and instead embrace a scientific holistic approach [10].

## 3. Sympathetic Nervous System as a Key Element of the Stress Response System

Vertebrate animals, including humans, try to accommodate to stressful situations through the allostatic action of their stress response system. This dynamic system is composed principally of a hormonal element, the hypothalamic-pituitary-adrenal axis, and a neural component, the sympathetic and parasympathetic nervous systems [1, 11]. Both parts of the stress response system are closely integrated and complementary. This review will focus on the sympathetic nervous system, since riotous activation of this system in FM could theoretically convert distress into pain.

The sympathetic nervous system is the “accelerating” part of the autonomic nervous system. Its antagonistic calming counterpart is the parasympathetic nervous system. The autonomic nervous system is in charge of maintaining homeostasis through the harmonious and opposing actions of these two branches. This balance maintains internal organ functions, and also maintains the vital signs. 

Structures in the central nervous system that generate patterns of autonomic response have been identified. These pattern generators are located at multiple levels in the central nervous system, including the hypothalamus and locus coeruleus, and they can be combined in temporal and spatial patterns to subserve a wide range of behavioral needs [11]. 

The peripheral sympathetic nervous system consists of a rich neural network that originates in the thoraco-lumbar section of the spinal column. The preganglionic sympathetic neurons have cell bodies within the horns of the spinal gray matter. Nerve fibers from these cell bodies extend to three types of ganglia grouped as paired paravertebral sympathetic chains, various unpaired distal plexuses, and terminal or collateral ganglia near the target organ. Sympathetic postganglionic fibers innervate the trunk and limbs via the spinal nerves. The sympathetic distribution to the head and neck comes from the three ganglia of the cervical sympathetic chain. The unpaired prevertebral ganglia reside in the abdomen and pelvis, anterior to the vertebral column, and are the celiac, superior mesenteric, aorticrenal, and inferior mesenteric ganglia. Postganglionic fibers arising from synaptic links of the upper thoracic sympathetic fibers in the vertebral ganglia form the terminal cardiac, esophageal, and pulmonary plexuses. The postganglionic fibers from the celiac, superior, and inferior mesenteric plexuses innervate the viscera of the abdomen and pelvis. The adrenal medulla and other chromaffin tissue are homologous to the sympathetic ganglia.

The sympathetic network is highly interconnected. Nerve trunks unite the paravertebral ganglia to each other, so that, in response to physical and/or mental stressors, sympathetic activation induces a diffuse amplified response that puts the whole organism in a state of high alert, ready to fight or flight. Sympathetic hyperactivity induces nervousness, pupil dilation, tachycardia, skin and visceral vasoconstriction, and muscle vasodilation in addition to many other reactions [12]. 

Catecholamines (norepinephrine and epinephrine) are the main postganglionic sympathetic neurotransmitters acting on a delicate dampening synaptic structure, the adrenergic receptor. Adrenergic receptors are G coupled proteins that are fundamental in maintaining homeostasis. These receptors are broadly classified as alpha and beta receptors. Alpha adrenergic receptors are more directly related to vasoconstriction whereas beta receptors increase cardiac output and vasodilation. Adrenergic receptors are expressed not only in the sympathetic nervous system, but also in virtually every cell type of the body. Lymphocytes and platelets have adrenergic receptors that are responsive to catecholamines. Hence, sympathetic activation may have coagulation and immune consequences [13]. 

Sympathetic nervous system dynamics are difficult to assess. Static blood or urinary measurements of catecholamines are unable to follow the constantly changing activity of the sympathetic nervous system. Fortunately, there is a new dynamic method based on computers that is able to estimate sympathetic and parasympathetic influence on heart rhythms. This method is called heart rate variability (HRV) analysis. As will be discussed later, diverse HRV studies have found abnormalities of the sympathetic nervous system in FM patients.

## 4. The Autonomic Nervous System as a Complex Adaptive System

Science is an ever changing discipline. The current linear-reductionist medical model appears unable to understand FM and similar maladies. Complexity is a new scientific paradigm that will probably have an impact on the practice of medicine. Complexity originated from the computer's awesome ability to perform calculations that are far beyond the capability of the human brain. Complexity recognizes that the universe is full of complex systems that work in an apparent disorderly way in an effort to adapt to the changing environment. In these complex systems, the magnitude of the response is not proportional to the intensity of the stimulus, therefore, linear algorithms are futile. Similarly, for complex systems, the whole is different from the sum of its parts, therefore, reductionist approaches are useless. The only way to understand a complex system is with a holistic approach, viewing the system dynamics in its entirety and assessing its adaptation to the environment. Therefore, holism has now scientific basis. Healthy complex systems have disorderly elastic behavior. If a complex system evolves into an orderly predictable behavior, it suffers degradation and ultimately dies. 

Complexity may change some medical dogmas. The current medical view proposes that disease appears when there is disorder in the function of the body. Complexity proposes the contrary: disorder is healthy and uniformity leads to disease. Existing linear clinical-pathologic correlations view structural damage as the essence of disease. New paradigms propose that disease is dysfunction. 

Complexity science paradigms may help us to understand FM. The autonomic nervous system can be viewed as a complex adaptive system. It is a hierarchic and decentralized self-regulatory system, from cells to neurons and from neurons to nerve system. Each level upholds a measure of independence while contributing to a higher level of organization. As will be discussed later, autonomic alterations in FM suggest degradation of this adaptive system, with the presence of rigid sympathetic hyperactivity. From a philosophical perspective, FM can be viewed as a failed attempt of our main complex adaptive system to accommodate to a hostile environment [14].

## 5. Animal Models Linking the Development of Sympathetic Pain to Physical or Emotional Trauma and to Different Types of Infections

Animal models demonstrate that physical trauma and distress can lead to chronic pain through aberrant sympathetic transmission. Sciatic nerve ligation is a standard model used to study neuropathic pain [15]. In rats, sciatic nerve injury induces abnormal connections between the sympathetic nervous system and the nociceptive system. These short-circuits take place in the rich synaptic paravertebral nodules called dorsal root ganglia. Under normal circumstances, there is not significant sympathetic innervation of dorsal root ganglia. Things change dramatically after nerve injury: there is sympathetic fiber sprouting via nerve growth factor over-expression. In such instances, catecholamines or sympathetic activity are able to induce pain and hyperalgesia [16]. 

Dorsal root ganglia may also act as a sanctuary for infective agents. Herpes virus may lay dormant for years in these sites. Upon reactivation, herpes may induce dorsal root ganglia neuroplasticity. Postherpetic neuralgia may be the resulting painful manifestation. A similar mechanism may operate in cases of chronic Lyme's disease [17].

Sodium channels located in dorsal root ganglia are the molecular gatekeepers of pain detection at peripheral nociceptors. Nine sodium channel subunits have been identified (Nav1.1–Nav1.9), each with a unique central and peripheral nervous system distribution. An isoform (Nav1.7) encoded in gene SCN9A of chromosome 2q24.3 is predominantly expressed in the dorsal root ganglia pain-sensing neurons and sympathetic ganglia neurons. A single Nav1.7 mutation (L858H) induces electrical hyperactivity of sensory neurons in dorsal root ganglia and, at the same time, produces hyporeactivity of sympathetic ganglia neurons. Several sodium “channelopathies” have been associated to rare painful dysautonomic syndromes such as primary erythermalgia and paroxysmal extreme pain disorder (formerly familial rectal pain syndrome) [18]. 

Animal models also show how distress can lead to pain. In rats, unpredictable noise can induce catecholamine-mediated chronic mechanical hyperalgesia [19]. 

These animal models illustrate how physical or emotional distress, as well as different types of infections, can induce sympathetically-dependent chronic pain conditions. Emerging evidence suggests that similar mechanisms may be operative in FM.

## 6. Physical and Emotional Distress in FM

Both physical and emotional stressors are frequent FM triggers. Bodily trauma, particularly a whiplash injury during automobile accident, is a recognized FM trigger [3, 4]. Also, different types of infections have been associated with the development of FM [20]. Agents implicated include viruses (e.g., hepatitis C, HIV, herpes) and Borrelia, the latter is the infecting agent in cases of Lyme's disease [21]. Women with FM have increased incidence of prior sexual or physical abuse [22]. 

Moreover, FM patients are often immersed in a stressful lifestyle. A prospective investigation found that the development of this illness was associated to workplace bullying, high workload, and low decision-making possibilities [23]. Anxiety and depression are frequent FM companions. Furthermore, many FM patients appear to have created their own “lifestyle stress” by physically or mentally overexerting themselves, being too perfectionist or overcommitted at work, or engaging in disproportionate self-sacrificing behavior [6].

## 7. Genetic and Clinical Data Suggesting That FM Is a Sympathetically Maintained Neuropathic Pain Syndrome

**Figure 1:**
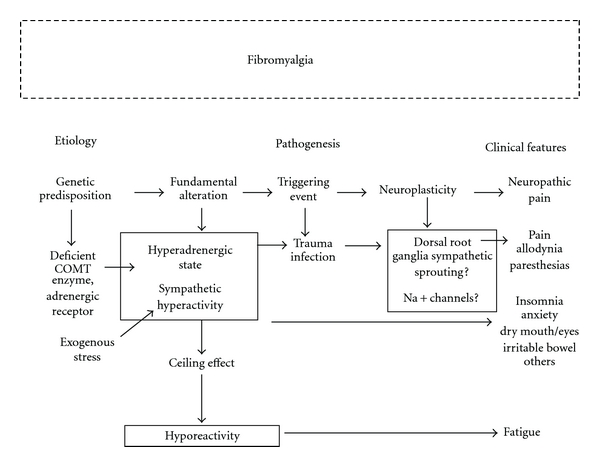
Theoretical etiopathogenetic mechanisms in fibromyalgia. See text for details. COMT: catechol-*O*-methyltransferase.

Different groups of investigators have described sympathetic nervous system dysfunction in patients suffering from FM. Most investigations used HRV analysis as a probing tool. From the literature review, a clear pattern of sympathetic abnormalities in FM emerges: basal sympathetic hyperactivity accompanied by blunted sympathetic response to different types of stressors. HRV alterations suggestive of sympathetic dysfunction are perhaps the most consistent alteration described so far in FM. We have proposed that this abnormal sympathetic behavior may explain all symptoms and that FM is a sympathetically maintained neuropathic pain syndrome [1]. Mechanisms whereby sympathetic dysfunction leads to fibromyalgia multisystem features are outlined in Figure 1.

The International Association for the Study of Pain defines neuropathic pain as “Pain initiated or caused by a primary lesion or dysfunction in the nervous system.” Following linear algorithms, a group of experts from the same society recently redefined neuropathic pain as “pain arising as the direct consequence of a lesion or disease affecting the somatosensory system,” and a grading system of “definite,” “probable,” and “possible” neuropathic pain was introduced. This linear redefinition emphasizes “damage” over “dysfunction” as the essence of disease [24]. As already stated, in contrast to this point of view, new scientific paradigms suggest that disease is dysfunction.

We propose that FM is a neuropathic pain syndrome based on the following three arguments.

1. FM is a stimulus-independent pain state. There is no structural damage that could explain the pain intensity.
2. The presence of allodynia as an essential feature of FM.
3. The presence of paresthesias as a distinctive feature of FM.

It seems difficult to ascribe an etiology other than neuropathic pain to a syndrome with such characteristics. 

Among neuropathic pain syndromes, we propose that FM pain is sympathetically maintained based on the following three issues.

1. The high frequency of physical or psychological trauma as a triggering event [2–4].
2. The signs of ongoing sympathetic hyperactivity [1].
3. A double-blind study showing that norepinephrine injections rekindle FM pain [25].

Genetic studies support the concept of FM as a sympathetically maintained pain syndrome. Catechol-*O*-methyltransferase (COMT) is the enzyme in charge of clearing catecholamines from the system. In healthy individuals, pain perception is related to COMT gene haplotypes linked to a defective catecholamine clearing enzyme. A prospective study demonstrated that healthy individuals with such gene haplotypes are prone to develop chronic pain syndromes, such as temporomandibular joint disorder [26]. Several groups of investigators have shown COMT gene alterations in FM patients [27, 28]. It should be noted, however, that this association was not found in a study of individuals with chronic widespread pain [29]. The reason for this discordance remains to be explained. Additionally, people suffering from FM have gene polymorphisms associated to defective adrenergic receptors [30]. 

The concept of FM as a sympathetically maintained neuropathic pain syndrome may offer new avenues for research. Firstly, this concept validates FM symptoms. Few physicians would doubt the validity of postherpetic neuralgia, even in the absence of ostensible tissue damage. Additionally, the fruitful animal and human research on the pathogenesis of neuropathic pain and sympathetic pain can be applied to the study of FM. 

Recent genetic studies suggest that severe FM may be a sodium channelopathy. When compared to healthy controls, Mexican women with FM have different gene polymorphisms of sodium channels located in dorsal root ganglia and in sympathetic ganglia [31]. This new finding may have therapeutic implications. Sodium channel blockers may become useful therapeutic agents for FM. Currently available sodium channel blockers are weak and nonspecific. Selective tetrodotoxin-resistant channel blockers are being developed and may in the future constitute an important therapeutic option for FM pain [18].

## 8. Conclusions

FM can be viewed as a failed attempt of our main complex adaptive system to accommodate to a hostile environment. A disease in which distress is transformed into pain through sympathetic system rigidity. Proposed mechanisms whereby this transformation takes place are summarized in Figure 1. Certain COMT or adrenergic receptor gene polymorphisms predispose to persistent hyperadrenergic state. Physical or emotional trauma may act as triggering events. Different types of infections may also set off the disease. Such stressors are confronted by the main element of the stress response system, the sympathetic nervous system. Resulting chronic sympathetic hyperactivity leads to aberrant dorsal root ganglia neuroplasticity, establishing abnormal connections between the sympathetic nervous system and the nociceptive system. Altered expression of sodium channels takes place. A clinical syndrome of sympathetically maintained neuropathic pain syndrome develops. Sympathetic hyperactivity may also explain other FM symptoms such as anxiety, insomnia, and irritable bowel. Sympathetic hyporeactivity to stress may explain the constant fatigue. 

This fibromyalgia dysautonomic paradigm demands a holistic type of therapy in an effort to regain complexity of our main adaptive system and, thus, improving basal sympathetic tone [10]. Specific sodium channel blockers may become valuable agents to relieve the most vexing manifestation of FM: chronic widespread pain.
